# Cognitive Behavioral Therapy App, Resting State Functional Connectivity, and Anxiety

**DOI:** 10.1001/jamanetworkopen.2025.24498

**Published:** 2025-07-31

**Authors:** Abhishek Jaywant, Jennifer N. Bress, Charles J. Lynch, Zareen Mir, Maddy M. Schier, Hussain Bukhari, Holland Brown, Avital Falk, Shannon Bennett, Roy H. Perlis, Conor Liston, Francis S. Lee, Faith M. Gunning

**Affiliations:** 1 Department of Psychiatry, Weill Cornell Medicine, New York, New York; 2 Department of Rehabilitation Medicine, Weill Cornell Medicine, New York, New York; 3 Massachusetts General Hospital, Harvard Medical School, Boston

## Abstract

This cohort study examines the association between baseline resting state functional connectivity and improvement in anxiety after use of a mobile cognitive behavior therapy application.

## Introduction

Anxiety disorders are prevalent among young adults. Cognitive-behavioral therapy (CBT) is an effective treatment, but many individuals have difficulty accessing CBT due to cost and limited availability.^1^ Digital applications (hereafter, *apps*) may increase accessibility. We recently showed that use of a mobile CBT app (Maya)^2^ was associated with reductions in anxiety symptoms in young adults.^3^

However, only 50% to 60% of individuals respond to CBT.^4^ Identifying brain mechanisms associated with differential treatment response could advance understanding of what makes patients likely to respond to CBT. We examined whether baseline resting state functional connectivity (rsFC) was associated with improvement in anxiety after use of the app.

## Methods

This cohort study was a secondary analysis of a published trial^3^ that recruited individuals age 18 to 25 years with a diagnosed anxiety disorder between August 2021 and November 2022. All participants provided written informed consent. All procedures were approved by the Weill Cornell Medicine institutional review board. This report follows STROBE reporting guidelines. Data on race and ethnicity are included in this study to provide information on the generalizability of the findings.

The app included interactive modules focused on identifying emotions, exposure, reframing negative thoughts, and relaxation.^3^ Participants completed two 20-minute sessions per week for 6 weeks in addition to meeting weekly with research staff to assess mood and adherence.

Participants were invited to undergo optional magnetic resonance imaging (MRI) prior to the intervention. MRI acquisition methods are described in the eMethods in Supplement 1. In this exploratory analysis, we tested the association between rsFC in 9 a priori defined regions, separately in the left and right hemisphere, previously associated with anxiety symptoms and/or response to CBT (eTable in Supplement 1). The primary outcome was change in the Hamilton Anxiety Rating Scale (HAM-A), a clinician-interview measure of anxiety symptom severity. We computed multivariable general linear models evaluating the association between connectivity for pairs of regions of interest and HAM-A change while covarying for age and baseline HAM-A score (72 total region of interest pairs tested, 36 in each hemisphere). We chose an α = .01 and computed a Benjamini-Hochberg false discovery rate (FDR) correction (*q* < .05).

## Results

A total of 32 participants (26 female [81%]; mean [SD] age, 23.4 [1.9] years) completed MRI and were included in this analysis. Participants had moderate anxiety at baseline (mean [SD] HAM-A score, 16.1 [6.5]), and exhibited a mean improvement in anxiety of 7.4 points (95% CI, 4.9-9.9 points) on the HAM-A (Table).

**Table.** Demographic and Clinical Characteristics of Participants

Characteristics	Participants, No. (%)		Standardized mean difference
	Completed optional MRI (n = 32)	Did not complete optional MRI (n = 25)	
Age, mean (SD), y	23.4 (1.9)	23.0 (2.1)	0.19
Gender			
Female	26 (81)	17 (71)	NA
Male	6 (19)	5 (21)	NA
Nonbinary	0	2 (8)	NA
Race			
American Indian	1 (3)	0	NA
Asian	13 (41)	7 (28)	NA
Black or African American	2 (6)	2 (8)	NA
White	13 (41)	12 (48)	NA
More than 1 race	1 (3)	1 (4)	NA
Unknown or not reported	1 (3)	1 (4)	NA
Other^a^	1 (3)	2 (8)	NA
Ethnicity			
Hispanic or Latino	3 (9)	2 (8)	NA
Not Hispanic or Latino	27 (84)	22 (88)	NA
Not reported	2 (6)	1 (4)	NA
Cognitive behavioral therapy application sessions completed, mean (SD)	10.9 (2.2)	10.8 (2.2)	0.04
Baseline Hamilton Anxiety Rating Scale score, mean (SD)	16.1 (6.5)	13.4 (6.5)	0.42

Abbreviations: MRI, magnetic resonance imaging; NA, not applicable.

^a^ Other race refers to racial selection as other than Asian, Black or African American, American Indian or Alaska Native, Native Hawaiian or Other Pacific Islander, White, or more than one race designation.

Lower baseline connectivity between the left anterior insula and left dorsolateral prefrontal cortex was associated with greater improvement in HAM-A score after use of the CBT app, although this association did not survive the more stringent FDR correction (β = −0.39; 95% CI, −0.61 to −0.16; *t* = −3.47; uncorrected *P* = .002; FDR-corrected *P* = .12) (Figure). No other associations were statistically significant after correction.

**Figure.**
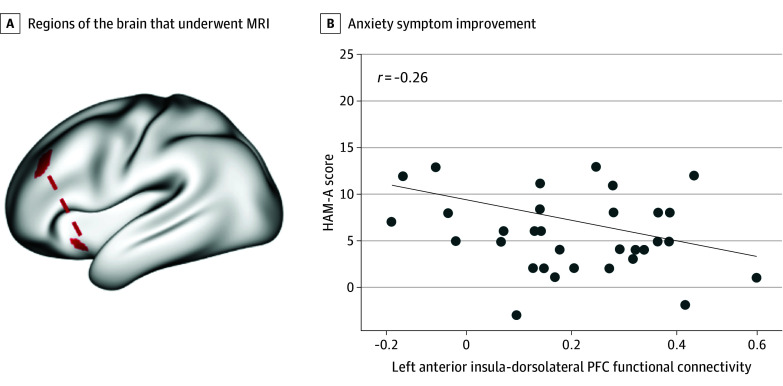
Unadjusted Association Between Improvement on the Hamilton Anxiety Rating Scale (HAM-A) and Resting State Functional Connectivity A, Surface view showing left anterior insula-dorsolateral prefrontal cortex region of interest pair. B, Graph shows association between improvement on HAM-A and functional connectivity in the left anterior insula (*x* = −32.5, *y* = 17.2, and *z* = −7.8) and the left dorsolateral prefrontal cortex/middle frontal gyrus (*x* = −35.7, *y* = 33.1, and *z* = 32).

## Discussion

This cohort study found that lower baseline rsFC between the anterior insula of the salience network and the dorsolateral prefrontal cortex was associated with greater symptomatic improvement in anxiety following use of a mobile, self-guided CBT app. This result requires replication given that it did not survive stringent FDR correction. The anterior insula is involved in vigilance to emotionally relevant information and interoceptive processing.^5^ These exploratory results suggests that lower insula-dorsolateral prefrontal cortex connectivity, possibly reflecting more adaptive emotional processing, is associated with greater improvement of anxiety symptoms with app-delivered CBT skills. This extends prior work that suggests an association between functional connectivity of the salience network and response to face-to-face CBT in adolescents.^6^

Our sample was predominantly female and was not racially representative of the general US population, which may limit generalizability. Still, these exploratory findings provide a foundation for future preregistered, hypothesis-driven work to understand the brain network predictors of digital mental health interventions. This approach could inform the development of effective digital interventions for young anxious adults by informing the tailoring of treatment to patterns of brain network connectivity.

## Notes

Supplement 1. **eAppendix.** Supplemental methods **eTable.** ROI names, network assignments, and centroid coordinates for ROIs used in analysis
eReferences

Supplement 2.
Data Sharing Statement
