# Supplementary material for: Cognitive Behavioral Therapy App, Resting State Functional Connectivity, and Anxiety
Source: JAMA Netw Open. 2025 Jul 31;8(7):e2524498. doi: 10.1001/jamanetworkopen.2025.24498 (PMC12314715; doi:10.1001/jamanetworkopen.2025.24498)
Supplement: Supplement 1. — eAppendix. Supplemental methods eTable. ROI names, network assignments, and centroid coordinates for ROIs used in analysis eReferences [file jamanetwopen-e2524498-s001.pdf]

Supplemental Online Content

Jaywant A, Bress JN, Lynch CJ, et al. Use of a cognitive behavioral therapy application and reduction in anxiety in young adults. *JAMA Netw Open.* 2025;8(8):e2524498. doi:10.1001/jamanetworkopen.2025.24498

eAppendix. Supplemental methods

eTable. ROI names, network assignments, and centroid coordinates for ROIs used in analysis

eReferences

This supplemental material has been provided by the authors to give readers additional information about their work.

© 2025 Jaywant A et al. *JAMA Network Open*.

eAppendix. Supplemental methods

Participant Inclusion and Exclusion Criteria

Participants were recruited from the greater New York City metropolitan area using online advertisements, local university and college listservs, and flyers posted at local universities and colleges. All participants had a primary diagnosis of an anxiety disorder as assessed by the Anxiety Disorder Interview Schedule for DSM-5[1] with a clinical severity rating of 4 or greater. Prospective participants were excluded if they were already participating in CBT, had initiated or modified dosage of one or more psychotropic medications within the past 12 weeks, or endorsed active suicidal ideation. As this study was conducted during the Covid-19 pandemic, some participants reported that they chose not to participate in the MRI due to personal discomfort around social interactions. Others did not complete the MRI because of contraindications, self-reported claustrophobia, or personal preference. Two subjects were excluded from analysis because of excessive motion (>50% of volumes censored for framewise displacement >0.3).

Detailed neuroimaging preprocessing and denoising procedure.

MRI data were collected using a 3T Siemens Prisma scanner with a 32-channel head coil. Anatomical images included a 3D T1-weighted MPRAGE sequence (TR = 2.4s, TE = 2.22ms, slice thickness = 0.8mm, flip angle = 8°) and T2-weighted FLAIR SPACE (TR = 6.6s, TE = 0.39s, slice thickness = 0.98mm, flip angle = 120°). Anatomical preprocessing included the following steps using the Pre-Freesurfer, Freesurfer, and Post-Freesurfer scripts of the Human Connectome Project processing pipeline: cropping of T1- and T2- weighted images to a smaller field of view; co-registration using FSL epi_reg[2]; correction for intensity inhomogeneities; co-registration to the standard MNI152 template using a six degrees of freedom FLIRT transformation; generation of cortical surfaces including the pial, white, and midthickness boundaries using Freesurfer 6.0[3] "recon-all" pipeline with the "-T2pial" option; registration to fs_LR space; and resampling of the resolution to 32k vertices using Connectome Workbench[4].

Functional images were collected using a multiband multiecho T2*-weighted EPI sequence with TR = 1.33s, slice thickness = 2.5mm, flip angle = 67°, four echoes (12.6ms, 29.5ms, 46.4ms, 63.3ms), and a total of 960 volumes acquired from two 11-minute runs. We used a multiecho acquisition sequence because previous work has shown that multiecho fMRI has significant improvements over traditional single-echo fMRI in enhancing signal, improving reliability, and reducing noise by better accounting for non-BOLD related artifacts[5]

Resting state fMRI preprocessing, denoising, and analysis followed an in-house multiecho fMRI pipeline that has previously been described elsewhere (see Lynch et al., above). Preprocessing and denoising of resting state fMRI data was completed separately for each of the two runs for each participant. Preprocessing steps included averaging of a single-band reference image for each of the four echoes; alignment and co-registration of this averaged single-band reference image to the T1-weighted anatomical image with simultaneous correction for spatial distortions using FSL topup and epi_reg; refinement of this registration with Freesurfer's bbregister algorithm; and slice timing correction using FSL's slicetimer. These steps were implemented as one concatenated step using a spline warp. Echoes were then combined at each timepoint and registered to the single-band reference image that was estimated with FSL's MCFLIRT tool with a 4-stage sinc optimization.

Denoising used the Tedana program[6], which optimally combines the multiecho data and uses principal component analysis to isolate spatial components that are BOLD-like and non-BOLD like and discards non-BOLD (noise) components. Tedana outputs were manually reviewed to ensure accurate classification of signal and noise components following Griffanti et al[7]. We then performed mean gray matter time-series regression to remove spatially diffuse noise.

The following steps were taken after denoising: mapping of the fMRI time-series data to the midthickness surface using the CIFTI format in Connectome Workbench; demeaning; concatenating the two runs of data; spatial smoothing with geodesic Gaussian kernels (sigma = 2.55mm); censoring volumes with framewise displacement > 0.3mm; parcellation using the Gordon 333 region cortical atlas[8] and Seitzman atlas of 61 subcortical regions[9]; and Fisher's r-to-z transformation.

© 2025 Jaywant A et al. *JAMA Network Open*.

We subsequently selected nine out of the 394 ROIs in the left hemisphere and the corresponding nine ROIs in the right hemisphere. We focused on ROIs within the ECN (lateral prefrontal cortex and caudate), salience network, amygdala, and ventral striatum that have previously demonstrated association with anxiety in adolescents and young adults and response to traditional CBT. Specifically, we selected and calculated connectivity in the following ROIs: dorsal anterior cingulate cortex, anterior insula, and lateral prefrontal parcels of the salience network; three parcels encompassing the dorsolateral and middle prefrontal cortex of the ECN; one parcel encompassing a portion of the caudate belonging to the ECN; one parcel encompassing the ventral striatum of the reward network; and one parcel encompassing the amygdala. Network assignment of ROIs was based on Perino et al.[10] for the Gordon cortical atlas, and on Seitzman et al. for the subcortical atlas.

© 2025 Jaywant A et al. *JAMA Network Open*.

eTable. **ROI names, network assignments, and centroid coordinates for ROIs used in analysis**

Left Hemisphere	Network	X	Y	Z
DLPFC	Frontoparietal	-38.1	48.8	10.5
Anterior Cingulate	Salience	-10	33.9	21.5
Anterior Insula	Salience	-32.5	17.2	-7.8
DLPFC	Frontoparietal	-40.2	23.6	23.3
Lateral PFC	Frontoparietal	-28.6	50.9	10.1
DLPFC (middle frontal gyrus)	Salience	-35.7	33.1	32
Amygdala	Reward	-20.3	-2.27	-22.21
Ventral Striatum	Reward	-12.49	17.05	-4.49
Caudate	Frontoparietal	-15.19	-1.5	18.84
**Right Hemisphere**	**Network**	**X**	**Y**	**Z**
Anterior Cingulate	Salience	8.4	34.7	22.6
DLPFC	Frontoparietal	36.8	37.8	13.1
Anterior Insula	Salience	30.6	22.8	-4.7
DLPFC	Frontoparietal	41.8	29.1	21.6
DLPFC (middle frontal gyrus)	Salience	31.3	39.7	25.6
DLPFC	Frontoparietal	30.9	52.2	9.9
Amygdala	Reward	19.51	-1.85	-23.11
Ventral Striatum	Reward	12.66	17.32	-5.06
Caudate	Frontoparietal	14.15	-1.19	18.18

© 2025 Jaywant A et al. *JAMA Network Open*.

eReferences

1. Brown TA, Barlow DH. *Anxiety and Related Disorders Interview Schedule for DSM-5 (ADIS-5): Client Interview Schedule*. Adult version. Oxford University Press; 2014.
2. Jenkinson M, Beckmann CF, Behrens TEJ, Woolrich MW, Smith SM. FSL. *NeuroImage*. 2012;62(2):782-790. doi:10.1016/j.neuroimage.2011.09.015
3. Fischl B. FreeSurfer. *NeuroImage*. 2012;62(2):774-781. doi:10.1016/j.neuroimage.2012.01.021
4. Marcus DS, Harwell J, Olsen T, et al. Informatics and Data Mining Tools and Strategies for the Human Connectome Project. *Front Neuroinform*. 2011;5. doi:10.3389/fninf.2011.00004
5. Lynch CJ, Power JD, Scult MA, Dubin M, Gunning FM, Liston C. Rapid Precision Functional Mapping of Individuals Using Multi-Echo fMRI. *Cell Reports*. 2020;33(12):108540. doi:10.1016/j.celrep.2020.108540
6. DuPre E, Salo T, Ahmed Z, et al. TE-dependent analysis of multi-echo fMRI with tedana. *JOSS*. 2021;6(66):3669. doi:10.21105/joss.03669
7. Griffanti L, Douaud G, Bijsterbosch J, et al. Hand classification of fMRI ICA noise components. *NeuroImage*. 2017;154:188-205. doi:10.1016/j.neuroimage.2016.12.036
8. Gordon EM, Laumann TO, Adeyemo B, Huckins JF, Kelley WM, Petersen SE. Generation and Evaluation of a Cortical Area Parcellation from Resting-State Correlations. *Cereb Cortex*. 2016;26(1):288-303. doi:10.1093/cercor/bhu239
9. Seitzman BA, Gratton C, Marek S, et al. A set of functionally-defined brain regions with improved representation of the subcortex and cerebellum. *NeuroImage*. 2020;206:116290. doi:10.1016/j.neuroimage.2019.116290
10. Perino MT, Myers MJ, Wheelock MD, et al. Whole-Brain Resting-State Functional Connectivity Patterns Associated With Pediatric Anxiety and Involuntary Attention Capture. *Biological Psychiatry Global Open Science*. 2021;1(3):229-238. doi:10.1016/j.bpsgos.2021.05.007

© 2025 Jaywant A et al. *JAMA Network Open*.
