# Supplementary material for: Cognitive Behavioral Therapy App, Resting State Functional Connectivity, and Anxiety
Source: JAMA Netw Open. 2025 Jul 31;8(7):e2524498. doi: 10.1001/jamanetworkopen.2025.24498 (PMC12314715; doi:10.1001/jamanetworkopen.2025.24498)
Supplement: Supplement 2. — Data Sharing Statement [file jamanetwopen-e2524498-s002.pdf]

Data Sharing Statement

Jaywant. Use of a Cognitive Behavioral Therapy Application and Reduction in Anxiety in Young Adults. *JAMA Netw Open*. Published August 01, 2025.
doi:10.1001/jamanetworkopen.2025.24498

Data
Data available: Yes
Data types: Deidentified participant data, Data dictionary
How to access data: All requests for data should be sent to Dr. Abhishek Jaywant at abj2006@med.cornell.edu
When available: With publication

Supporting Documents
Document types: Statistical/analytic code
How to access documents: Analytic code requests should be sent to Dr. Abhishek Jaywant at abj2006@med.cornell.edu
When available: With publication

Additional Information
Who can access the data: Researchers whose proposed use of the data has been approved by the study team and subject to a data use/sharing agreement.
Types of analyses: For any purpose, subject to approval of the study team.
Mechanisms of data availability: After approval of a proposal and with a signed data access agreement.
